# Multicenter GeoSentinel Analysis of Rickettsial Diseases in International Travelers, 1996–2008

**DOI:** 10.3201/eid1511.090677

**Published:** 2009-11

**Authors:** Mogens Jensenius, Xiaohong Davis, Frank von Sonnenburg, Eli Schwartz, Jay S. Keystone, Karin Leder, Rogelio Lopéz-Véléz, Eric Caumes, Jakob P. Cramer, Lin Chen, Philippe Parola, for the GeoSentinel Surveillance Network

**Affiliations:** Oslo University Hospital, Oslo, Norway (M. Jensenius); University of Oslo, Oslo (M. Jensenius); Centers for Disease Control and Prevention, Atlanta, Georgia, USA (X. Davis); University of Munich, Munich, Germany (F. von Sonnenburg); Chaim Sheba Medical Centre, Tel Hashomer, Israel (E. Schwartz); Toronto General Hospital, Toronto, Ontario, Canada (J.S. Keystone); Royal Melbourne Hospital, Parkville, Victoria, Australia (K. Leder); Ramón y Cajal Hospital, Madrid, Spain (R. Lopéz-Véléz); Hôpital Pitié-Salpêtrière, Paris, France (E. Caumes); Bernhard-Nocht-Clinic for Tropical Medicine, Hamburg, Germany (J.P. Cramer); Harvard Medical School, Boston, Massachusetts, USA (L. Chen); WHO Collaborative Center for Rickettsioses and Other Arthropod Borne Bacterial Diseases, Marseille, France (P. Parola); Hôpital Nord, Marseille (P. Parola); 1 Additional members of the GeoSentinal Surveillance Network who contributed data are listed at the end of this article.

**Keywords:** Travel, epidemiology, diagnosis, rickettsia, tick typhus, scrub typhus, Q fever, bartonella, GeoSentinel, research

## Abstract

Spotted fever group rickettsiosis acquired in sub-Saharan Africa was the most common rickettsial disease observed.

We investigated epidemiologic and clinical aspects of rickettsial diseases in 280 international travelers reported to the GeoSentinel surveillance Network during 1996–2008. Of these 280 travelers, 231 (82.5%) had spotted fever (SFG) rickettsiosis, 16 (5.7%) scrub typhus, 11 (3.9%) Q fever, 10 (3.6%) typhus group (TG) rickettsiosis, 7 (2.5%) bartonellosis, 4 (1.4%) indeterminable SFG/TG rickettsiosis, and 1 (0.4%) human granulocytic anaplasmosis. One hundred ninety-seven (87.6%) SFG rickettsiosis cases were acquired in sub-Saharan Africa and were associated with higher age, male gender, travel to southern Africa, late summer season travel, and travel for tourism. More than 90% of patients with rickettsial disease were treated with doxycycline, 43 (15.4%) were hospitalized, and 4 had a complicated course, including 1 fatal case of scrub typhus encephalitis acquired in Thailand.

Rickettsial diseases are acute and potentially severe zoonotic infections caused by obligate intracellular, gram-negative bacteria belonging to the order Rickettsiales. The taxonomy of Rickettsiales is complex and continues to be updated, but currently the agents of rickettsial diseases are classified as belonging to 4 distinct genera: *Rickettsia* (including 2 biogroups: spotted fever group [SFG] rickettsiae with >10 species and typhus group [TG] rickettsiae with 2 species)*, Orientia* (*Orientia tsutsugamushi*, the agent of scrub typhus)*, Ehrlichia* (*Ehrlichia chaffeensis,* the agent of human monocytic ehrlichiosis), and *Anaplasma* (*Anaplasma phagocytophilium*, the agent of human granulocytic anaplasmosis). Diseases caused by *Rickettsia* and *Orientia* species are often collectively referred to as rickettsioses. *Coxiella burnetii*, the agent of Q fever, and *Bartonella* spp. were recently removed from the order Rickettsiales, but Q fever and bartonelloses are still frequently categorized as rickettsial diseases (*1*).

Rickettsial diseases are increasingly being recognized among international travelers (*2*). A recent study of ≈7,000 returnees with fever as a chief reason to seek medical care suggests that 2% of imported fevers are caused by rickettsioses and that 20% of these patients are hospitalized (*3*). Most cases are acquired in sub-Saharan Africa, where SFG rickettsioses are second only to malaria as the most commonly diagnosed diseases in returnees with systemic febrile illness (*4*). With few exceptions, however, our knowledge of the incidence rates, associated factors, signs, symptoms, and outcome of rickettsial diseases in travelers is rudimentary and mostly based on smaller case series. We report all cases of rickettsial diseases in returned travelers reported to the GeoSentinel Surveillance Network from June 1996 through December 2008.

## Materials and Methods

### Data Source

GeoSentinel is a global network aimed at surveying ill international travelers and was established in 1995 through the International Society of Travel Medicine and the Centers for Disease Control and Prevention. The 41 current GeoSentinel sites contribute clinician-based, anonymous information on all ill travelers seen that is entered in a structured query language database at a central data center. Data collected include demographic information, recent travel history, reason for travel, outpatient or inpatient status, whether the patient was seen during or after travel, whether the patient traveled independently versus organized travel (i.e., travel with a tour group), whether the patient had pretravel encounter with a healthcare provider, patient symptoms, and diagnosis. All GeoSentinel sites use the best reference diagnostic methods available in the country where the site is located. The diagnosis, which may be reported as confirmed, probable, or suspected, is chosen from a standardized list of >500 possible etiologic and syndromic diagnoses, 11 of which refer to rickettsial diseases. Patients may receive several diagnoses, some of which describe well-known complications of rickettsial diseases, e.g., acute renal failure, acute encephalitis, acute respiratory distress syndrome, and death (*3*,*4*). The GeoSentinel questionnaire does not contain queries about case management, but in December 2008 all sites were requested to estimate the percentage of their patients with rickettsial diseases who received treatment with antirickettsial drugs (e.g., doxycycline) during the study period.

### Inclusion Criteria and Definitions

**Figure 1:**
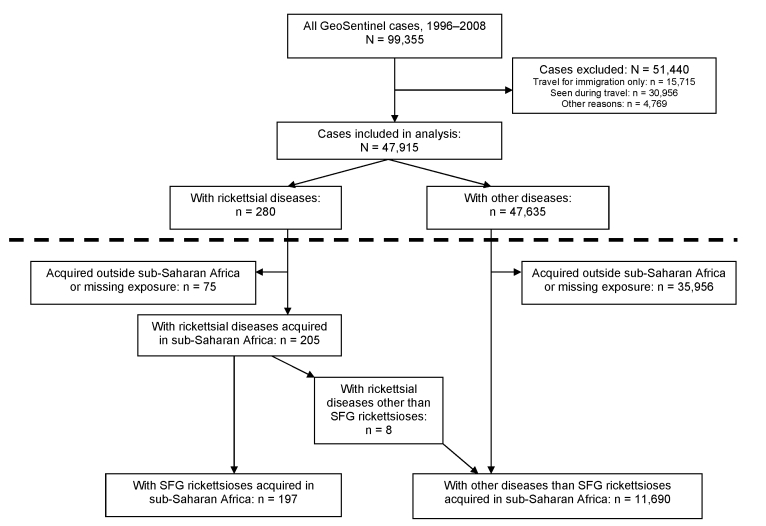
Study design with main study (above dotted line) and substudy (below dotted line) of spotted fever group (SFG) rickettsiosis acquired in sub-Saharan Africa, 1996–2008.

Data entered into the GeoSentinel database from June 1996 through December 2008 were reviewed. Only travelers seen with confirmed or probable diagnoses were included in the analysis. Cases of rickettsial diseases were defined according to the criteria mentioned below. A subanalysis, comparing SFG rickettsioses with other diseases in ill returnees from sub-Saharan Africa, was also performed (Figure 1).

A microbiologic diagnosis of recent rickettsial disease was in most cases based on immunofluorescence antibody (IFA) tests and, occasionally, on PCR and Western blot. Diagnostic criteria of recent infection by IFA tests were either a 4-fold increase of immunoglobulin (Ig) G or IgM titers in paired serum samples drawn >10 days apart, or elevated IgG and/or IgM titers in single samples consistent with recent infection as interpreted by the local microbiology laboratory. Because IFA tests cannot distinguish between species within the same *Rickettsia* biogroup, diseases caused by *Rickettsia* species were dichotomized as SFG rickettsiosis and TG rickettsiosis.

We included all cases of confirmed rickettsial disease defined as a traveler with pertinent travel history and clinical signs, and microbiologic test results supporting recent infection. For SFG rickettsiosis and scrub typhus we also included probable cases defined as a traveler with pertinent travel history and clinical signs including >1 inoculation eschars, and inconclusive or unavailable microbiologic test results. Cases were categorized in 9 disease groups: SFG rickettsiosis, TG rickettsiosis, indeterminate SFG/TG rickettsiosis (defined as cases in whom neither clinical signs nor microbiologic tests could distinguish between SFG and TG rickettsiosis), scrub typhus, ehrlichiosis, anaplasmosis, acute and chronic Q fever (based on the presence of antibodies to the 2 distinct antigenic phases of *C. burnetii* by IFA test [*5*]), and bartonellosis. Countries were grouped according to the United Nations region system, and for sub-Saharan Africa also in subregions; for clarification, the southern Africa subregion included Botswana, Lesotho, Namibia, South Africa, and Swaziland. Classification of reason for travel related to current illness was grouped into 4 categories: tourism, business, visiting friends and relatives, and other reason, which included studies or military, missionary, or foreign aid deployment.

### Statistical Analysis

All data were analyzed using SAS software, version 9 (SAS Institute, Cary, NC, USA). Comparison of travelers to sub-Saharan Africa with SFG rickettsioses with all other ill travelers to the same destination used the Student *t* test for continuous variables and Cochran-Mantel-Haenszel statistic for binary variables. The association between possible risk factors and a diagnosis of SFG rickettsioses acquired in sub-Saharan Africa was measured with odds ratios and 95% confidence intervals. Variables included in the univariate analysis were mean age, male gender, travel to southern Africa, travel from March through May, travel >1 week, no pretravel clinic visit, independent travel, and tourism as reason for travel. Variables with a p value <0.05 in the final multivariate logistic regression model were considered significant.

Proportionate morbidity for SFG rickettsiosis acquired in sub-Saharan Africa was defined as the number of cases as a proportion of all ill returned travelers from the region. Analysis of case reports over time was based on monthly proportionate morbidity, i.e., the number of patients with SFG rickettsiosis as a proportion of the number of all ill returned travelers attending any GeoSentinel site in that month. The monthly seasonality was based on aggregate data for that month over all years. The annual proportionate morbidity for SFG rickettsiosis acquired in southern Africa from 1999 through 2008 was estimated in a similar way based on relevant cases for each year. The Cochran-Armitage test was used for testing trend over years. This statistic tests for trend in binomial proportions across levels of a single factor or covariate and is appropriate for a contingency table where the response variable has 2 levels and the explanatory variable is ordinal. A 1-sided p value <0.05 would indicate a decreasing (with a negative statistic) or increasing (with a positive statistic) trend.

## Results

We identified 99,355 travelers who sought medical care at a GeoSentinel site during June 1996–December 2008. Among the 47,915 case-patients who met the study inclusion criteria, 280 (0.6%) had a diagnosis of rickettsial disease (Figure 1); among those having fever, the proportion was 211/13,763 (1.5%). Of travelers with rickettsial disease 231 (82.5%) had SFG rickettsioses, 16 (5.7%) scrub typhus, 11 (3.9%) acute Q fever, 10 (3.6%) TG rickettsioses**,** 7 (2.5%) bartonelloses, 4 (1.4%) indeterminate TG/SFG rickettsioses, and 1 (0.4%) human granulocytic anaplasmosis; there were no cases of chronic Q fever and ehrlichiosis. Cases were reported from 32 of the 41 active GeoSentinel sites: a total of 154 (54.9%) were reported from sites in Europe, 77 (27.5%) from North America, 17 (6.0%) from New Zealand/Australia, and 32 (11.6%) from Asia, including the Middle East. Most cases were associated with travel to sub-Saharan Africa (75.1%) (Table 1). A pretravel encounter with a healthcare provider was reported in 157 cases (58.4%). At least 90% of cases of rickettsial diseases were estimated to have been treated with antirickettsial drugs (doxycycline in most cases, occasionally a flouroquinolone) at the reporting sites during the study period.

**Table 1:** Travel destinations of 280 travelers with rickettsial diseases, by destination and disease, as reported to GeoSentinel, 1996–2008*

Destination	No. travelers						
	SFG rickettsiosis	TG rickettsiosis	Indeterminate SFG/TG rickettsiosis	Scrub typhus	Anaplasmosis	Acute Q fever	Bartonellosis
Western Europe	7	1			1	2	1
Eastern Europe			1				
North Africa	3						
Sub-Saharan Africa	197	1				5	1
Middle East	1					2	1
Northeast Asia	2	1				1	
South-central Asia	5	1	1	5			
Southeast Asia	3	6	2	9			1
Australia/New Zealand	1			1			
Oceania	1						
North America	1						
Central America	3						
Caribbean	1						3
South America							
Unknown	6			1		1	
Total	231	10	4	16	1	11	7

*SFG, spotted fever group; TG, typhus group.

### SFG Rickettsioses

Of the 231 cases of SFG rickettsioses (146 confirmed and 85 probable) a total of 136 (58.9%) were men; the mean age was 43.4 years (median 45 years, range 33–53 years). Tourists comprised 182 (78.8%), 28 (12.1%) had traveled for business, 6 (2.6%) were visiting friends and relatives, and 14 (6.1%) had traveled for other reasons. One hundred ninety-seven (87.6%) case-patients were infected in sub-Saharan Africa; South Africa (n = 135), Zimbabwe (n = 13), and Tanzania (n = 7) were the 3 most commonly reported countries of exposure (Table 1). The median time from travel to reporting to a GeoSentinel site was 8 days (range 4–12 days). Two case-patients, a 23-year-old French woman tourist to Mongolia in June 2008, and a 27-year-old Swiss woman student traveler to Corsica, France, in October 2008, were infected by *Rickettsia slovaca* confirmed by PCR and Western blot. There were no deaths among the travelers with SFG rickettsioses, but 22 (9.6%) were hospitalized, and multiorgan failure with acute respiratory distress syndrome developed in 2 Israeli men 47 and 73 years of age, respectively, after they traveled to India in October 2004.

**Figure 2:**
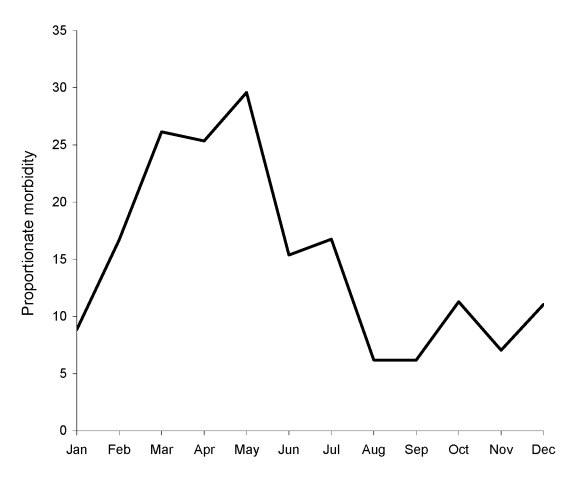
Monthly proportionate morbidity (no. cases/1,000 travelers) of spotted fever group rickettsiosis acquired in sub-Saharan Africa, 1996–2008.

**Figure 3:**
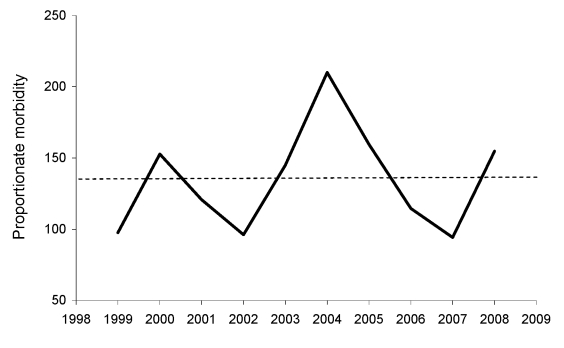
Annual proportionate morbidity (no. cases/1,000 travelers) of spotted fever group rickettsiosis acquired in southern Africa, 1996–2008. The dotted line indicates the mean value of 137/1,000 (13.7%).

Analysis of demographic and exposure variables for the 197 travelers to sub-Saharan Africa with SFG rickettsiosis compared with 11,690 travelers to the same region with other diagnoses is shown in Table 2. A multivariate logistic regression model was performed to analyze the effects on SFG rickettsiosis of risk factors including age, gender, travel to southern Africa, travel from March to May, travel duration longer than 7 days, no pretravel clinic visit, independent travel, and travel for tourism. Older age, male gender, travel to southern Africa, travel from March to May, and travel for tourism were found to be independently associated with SFG rickettsiosis. An analysis of monthly proportionate morbidity identified a peak of cases in March, April, and May (Figure 2). The proportionate morbidity of cases with SFG rickettsiosis in ill returnees from southern Africa from 1999 through 2008 was 139/1,017 (13.7%). The overall Cochran-Armitage trend test did not show statistically significant change (p = 0.877, by 2-sided trend test). As shown in Figure 3, the annual proportionate morbidity increased and decreased twice over the years and did not maintain a monotone increasing or decreasing pattern.

**Table 2:** Univariate and multivariate analyses of risk factors associated with SFG rickettsiosis in travelers to sub-Saharan Africa, 1996–2008*

Variable	Travelers with SFG rickettsiosis, N = 197†		Travelers without SFG rickettsiosis, N = 11,690†	Univariate association			Multivariate model‡	
				OR (95% CI)	p value		OR (95% CI)	p value
Mean age, y	43.9, n = 196		36.5, n = 11,608	–	<0.0001		1.02 (1.01–1.03)§	<0.0001
Male gender, no. (%)	115 (58.4), n = 197		6,105 (52.6), n = 11,599	1.26 (0.95–1.68)	0.11		1.40 (1.02–1.92)	0.035
Travel to southern Africa,¶ no. (%)	139 (70.6), n = 197		759 (6.5), n = 11,686	34.5 (25.18–47.28)	<0.0001		23.61 (16.86–33.07)	<0.0001
Travel in late summer,# no. (%)	89 (47.1), n = 189		4,220 (40.6), n = 10,402	1.30 (0.98–1.74)	0.07		1.57 (1.15–2.15)	0.005
Travel duration >7 d, no. (%)	173 (91.5), n = 189		9,661 (92.9), n = 10,402	0.83 (0.49–1.39)	0.48		0.67 (0.38–1.18)	0.164
No pretravel clinic visit, no. (%)	40 (21.7), n = 184		2,823 (26.5), n = 10,665	0.77 (0.54–1.10)	0.15		0.98 (0.66–1.44)	0.903
Independent travel,** no. (%)	34 (44.2), n = 77		4,100 (58.6), n = 6,993	0.56 (0.35–0.87)	0.01		0.83 (0.56–1.25)	0.373
Tourism as reason for travel, no. (%)	163 (82.7), n = 197		5,027 (43.0), n = 11,686	6.35 (4.38–9.21)	<0.0001		2.96 (1.97–4.45)	<0.0001

*SFG, spotted fever group; OR, odds ratio; CI, confidence interval. †n = number of travelers for whom this information was available. ‡This model included all variables considered at univariate level because of their clinical relevance. The Hosmer and Lemeshow goodness-of-fit test for this model is p = 0.575. §Odds ratio is for 1-y increase in age. ¶United Nations subregion comprising Botswana, Lesotho, Namibia, South Africa, and Swaziland. **#**March–May. **Independent travel was not formally collected by GeoSentinel until after May 2007.

### Other Rickettsial Diseases

The 10 travelers with TG rickettsiosis, of whom 6 were men, had a mean age of 28.0 years (median 25.5 years, range 20–50 years). Four case-patients were tourists, 2 were visiting friends and relatives, 1 was a business traveler, and 3 had traveled for other purposes. Southeast Asia, including 3 travelers infected in Indonesia, was the most common region of exposure (Table 1). The median time from travel to reporting a site was 6 days (range 3–14 days). Five patients were hospitalized and no complications were noted. One patient, a 23-year-old Japanese man visiting friends and relatives in Bali, Indonesia, had *R*. *typhi* infection confirmed by PCR.

Indeterminate SFG/TG rickettsiosis was diagnosed in 4 male tourists, 15, 50, 51, and 67 years of age, respectively. Two case-patients were infected in Southeast Asia (Table 1). No complications were recorded.

The 16 travelers with scrub typhus (5 confirmed and 11 probable), of whom 10 were men, had a mean age of 36.6 years (median 32.5 years; range 26–67 years). Nine patients were infected in Southeast Asia (including Thailand, n = 4) and 5 in south-central Asia (including India, n = 3). Twelve patients were tourists, 1 was visiting friends and relatives, and 3 had traveled for business. The median time from travel to reporting to a GeoSentinel site was 9 days (range 2–25 days). Six case-patients were hospitalized. Encephalitis developed in 2 Singaporean travelers: a 42-year-old man, a tourist infected in Thailand in December 2007, died, and a 51-year-old man infected in Taiwan while traveling on business in April 2008 survived.

An uncomplicated case of human granulocytic anaplasmosis was seen in August 2008 in a 32-year-old US woman tourist to the Netherlands. The diagnosis was based on detection of intracellular morulae.

The 11 travelers with acute Q fever, including 4 men, had a mean age of 53.0 years (median 59 years; range 20–64 years). Sub-Saharan Africa, including 2 case-patients infected in Tanzania, was the most common region of exposure (Table 1). Seven patients were tourists, 3 were business travelers, and 1 had traveled for other reasons. The median time from travel to reporting to a site was 21 days (range 5–52 days). Isolated fever (n = 7) and respiratory symptoms (n = 4) were the most common clinical manifestations. Four patients were hospitalized. All 11 patients with Q fever had uncomplicated clinical courses.

The 7 travelers with bartonellosis, including 3 males, had a mean age of 33.6 years (median 32 years, range 9–56 years). Three patients were infected in the Caribbean (Table 1). Four were tourists and 3 had traveled for other purposes. The median time from travel to reporting to a GeoSentinel site was 25 days (range 7–39 days). *Bartonella henselae* infection was diagnosed in all case-patients by serologic or immunohistochemical analysis. Six travelers had uncomplicated cat-scratch disease, and 1 had bacillary angiomatosis.

## Discussion

This study of 280 case-patients conducted over >12 years at 32 institutions on 5 continents represents the largest series of imported rickettsial diseases published to date. Noteworthy, SFG rickettsiosis was by far the most commonly diagnosed group of disease in our study. Numerous SFG rickettsioses occur throughout the world, the most important being Mediterranean spotted fever (with its variants Indian tick typhus, Astrakhan fever, and Israeli spotted fever) caused by *R*. *conorii conorii*, Rocky Mountain spotted fever caused by *R*. *rickettsii*, and African tick bite fever caused by *R*. *africae* (*1*). Some SFG rickettsioses may be accompanied by severe complications (*6**,**7*), as was exemplified by our 2 case-patients infected in India, possibly representing Indian tick typhus caused by *R. conorii indica* (*8*). Tick-borne lymphadenopathy (TIBOLA) caused by *R. slovaca*, a recently described entity associated with *Dermacentor* ticks and characterized by inoculation eschar, fatigue, and painful regional lymphadenitis (*9*), was diagnosed in 2 of our travelers. Being well documented across southern and central Europe, and especially so in children (*10*), our traveler infected in Mongolia represents the first documented case of TIBOLA acquired in Asia and illustrates how travel medicine may help identify new areas of endemicity for infectious diseases.

As shown here and by others (*11*), most cases of travel-associated SFG rickettsiosis are acquired in sub-Saharan Africa, and particularly in South Africa and neighboring countries. In the present series, as many as 13.7% of all healthcare-seeking returnees from southern Africa had a diagnosis of SFG rickettsiosis (Figure 3). Although several pathogenic SFG rickettsiae have been described in sub-Saharan Africa, including *R. conorii*, *R*. *siberica*, and *R*. *aeschlimannii* (*12*), 2 recent series evaluating 159 case-patients with species-specific tests, including PCR and Western blot, suggest that >99% of SFG rickettsioses diagnosed in international travelers to the region are caused by *R*. *africae* (*13*,*14*). In 1 of these reports, game hunting as reason for travel, travel to southern Africa, and travel during the summer season from November through April were found to be independent risk factors (*14*). If one assumes that most, if not all, of our 197 cases of SFG rickettsioses acquired in sub-Saharan Africa were de facto African tick bite fever, the present study suggests additional risk factors for this disease: male gender, higher age, travel during the late summer months of southern Africa (i.e., March–May; Figure 2), and travel for tourism. Although African tick bite fever is usually benign and self-limited, some patients may develop debilitating complications such as reactive arthritis, cranial and peripheral neuropathies, myocarditis, and neuropsychiatric symptoms, or experience a long-lasting convalescence, the latter phenomenon recently reported in elderly patients (*15*–*17*).

Our series comprises 10 cases of TG rickettsioses, all of which were considered to represent murine typhus caused by *R*. *typhi*, a bacteria transmitted from rodents to humans by rat fleas in many tropical and subtropical areas. Murine typhus is sometimes reported in returnees from the Mediterranean basin, Asia, and Africa; typical itineraries included travel to port cities or beach resorts (*18*,*19*). As in the present series, most infected travelers have mild disease with fever and constitutional symptoms, but complicated cases, including deaths, have been reported by others (*20*).

We also identified 16 cases of scrub typhus, a common disease in rural south and Southeast Asia and the Pacific. Before the present report, <30 cases of travel-associated scrub typhus had been published in the literature, and infection was predominantly acquired in Thailand and neighboring countries (*21*). The disease is transmitted by the bites of larval trombiculid mites (chiggers) that occur year round on grassy vegetation and is typically acquired by campers, trekkers, and visitors to rice paddies (*2*). Although most cases in travelers are mild and uncomplicated, scrub typhus may, as exemplified here, result in life-threatening encephalitis (*22*).

Human granulocytic anaplasmosis is a usually mild and nonspecific febrile illness commencing a few days after an *Ixodes* tick bite (*23*). It is endemic in North America and Europe with a geographic distribution that largely coincides with that of Lyme borreliosis. Human granulocytic anaplasmosis is rarely reported in international travelers but was recently diagnosed in an Austrian tourist visiting Slovenia (*24*). Our patient acquired the disease in 2008 in the Netherlands, where the first domestic case was reported in 1999 (*25*).

The ubiquitous Q fever, a zoonosis typically transmitted from domesticated mammals to humans by contaminated aerosols, is occasionally found in international travelers (*26*). Many infected travelers can recall visits to local farms and direct physical contact with domestic animals (*27*). All of our 11 case-patients had uncomplicated acute Q fever, the typical clinical sign in travelers, and most had a nonfocal febrile illness. However, it should be noted that acute Q fever may be severe and even fatal in travelers (*28*).

Bartonelloses are rarely reported in travelers but cases of Carrion disease caused by *Bartonella bacilliformis* have been seen after travel to South America (*29*,*30*). In the present series, we identified 7 case-patients likely having *B. henselae* infection, a ubiquitous condition associated with felines and their fleas and not previously reported in international travelers. Six of our patients had cat-scratch disease, a self-limiting disease characterized by cutaneous papules or pustules at the site at the inoculation site and an often long-lasting painful regional lymphadenitis, and 1 patient had bacillary angiomatosis, a potentially severe condition with vascular proliferation in the skin and internal organs (*31*).

With an overall incidence rate of 0.6% (and 1.5% of those travelers with fever), our analysis suggests that rickettsial diseases are uncommon in ill returnees. However, underdiagnosis is likely to be widespread, even at specialized institutions such as those associated with the GeoSentinel surveillance network. Few specific clinical signs exist in rickettsial diseases. Many cases have a nonfocal febrile disease of mild-to-moderate severity, accompanied by nonspecific results in routine blood tests (*32*). The inoculation eschar, a painless black skin lesion surrounded by a red halo that develops at the site of the infective tick or mite bite, is a useful diagnostic clue in SFG rickettsioses and scrub typhus, but may be absent in <40% of such cases (*14*). The available array of microbiologic diagnostic tests is another predicament in the management of rickettsial diseases. Although sensitive and specific techniques, such as PCR and culture, can be performed at reference centers, most cases worldwide are diagnosed by serologic analysis (*1*). The principal limitations with serologic analysis include a usually negative result in the acute phase when most patients first seek medical care, poor sensitivity in cases treated early with doxycycline, and an inability to distinguish between the various *Rickettsia* species caused by cross-reactions (*33*,*34*).

Some rickettsial diseases are potentially malignant, but severe complications developed in only a few patients in the present series, and only 1 traveler died. There may be several reasons for this favorable outcome, including a large percentage of benign African tick bite fever cases, and a widespread empiric use of antirickettsial drugs in cases of suspected rickettsial disease. It is noteworthy that rickettsial organisms are inherently resistant to many antimicrobial drugs commonly used as treatment for acute fevers, including the β-lactams, and treatment of choice is tetracyclines, in particular, doxycycline. Fluoroquinolones and newer macrolides may also be useful (*1*,*35*).

Our study had limitations similar to those in other GeoSentinel studies, including a possible selection bias towards complicated and unusual cases (*3*,*4*). Also, because rickettsial diseases, with the major exceptions of Q fever and bartonelloses, have short incubation periods (typically around 1 week) some cases are likely to manifest during travel and may be seen only by foreign healthcare providers. An analysis solely based on persons reporting posttravel is thus likely to underestimate the true incidence of rickettsial diseases in international travelers. However, because GeoSentinel currently has no site in sub-Saharan Africa, the prime destination for cases of travel-associated rickettsial diseases, we chose to exclude cases seen during travel from the analysis. Lastly, because most ill returnees seen at GeoSentinel sites have traveled to the tropics, we may have underestimated the incidence of rickettsial diseases typically acquired in temperate areas, including Rocky Mountain spotted fever, TIBOLA, and Mediterranean spotted fever.

In summary, the present study demonstrates the wide spectrum of rickettsial diseases that may be encountered in international travelers. Most infections are acquired in sub-Saharan Africa, where African tick bite fever is the predominate disease. The overall outcome is favorable but, because some rickettsial diseases may take a dire course, empirical treatment with an antirickettsial drug should always be considered whenever evaluating a traveler with an otherwise unexplained febrile disease who has recently returned from areas where these diseases are endemic.

## References

[1] Parola P, Paddock CD, Raoult D. Tick-borne rickettsioses around the world: emerging diseases challenging old concepts. Clin Microbiol Rev. 2005;18:719–56. 10.1128/CMR.18.4.719-756.2005 16223955 PMC1265907

[2] Jensenius M, Fournier PE, Raoult D. Rickettsioses and the international traveler. Clin Infect Dis. 2004;39:1493–9. 10.1086/425365 15546086

[3] Wilson ME, Weld LH, Boggild A, Keystone JS, Kain KC, von Sonnenburg F, Fever in returned travelers: results from the GeoSentinel Surveillance Network. Clin Infect Dis. 2007;44:1560–8. 10.1086/518173 17516399

[4] Freedman DO, Weld LH, Kozarsky PE, Fisk T, Robins R, von Sonnenburg F, Spectrum of disease and relation to place of exposure among ill returned travelers. N Engl J Med. 2006;354:119–30. 10.1056/NEJMoa051331 16407507

[5] Tissot-Dupont H, Raoult D. Q fever. Infect Dis Clin North Am. 2008;22:505–14. 10.1016/j.idc.2008.03.002 18755387

[6] Rutherford JS. Fatal spotted fever rickettsiosis, Kenya. Emerg Infect Dis. 2004;10:910–3. 15200829 10.3201/eid1005.030537 PMC3323220

[7] Chai JT, Eremeeva ME, Borland CD, Karas JA. Fatal Israeli spotted fever in a UK traveler to south Portugal. J Travel Med. 2008;15:122–3. 10.1111/j.1708-8305.2007.00179.x 18346246

[8] Parola P, Fenollar F, Badiaga S, Brouqui P, Raoult D. First documentation of *Rickettsia conorii* infection (strain Indian tick typhus) in a traveler. Emerg Infect Dis. 2001;7:909–10. 10.3201/eid0705.010527 11747712 PMC2631861

[9] Raoult D, Lakos A, Fenollar F, Beytout J, Brouqui P, Fournier PE. Spotless rickettsiosis caused by *Rickettsia slovaca* and associated with *Dermacentor* ticks. Clin Infect Dis. 2002;34:1331–6. 10.1086/340100 11981728

[10] Porta FS, Nieto EA, Creus BF, Espín TM, Casanova FJ, Sala IS, Tick-borne lymphadenopathy: a new infectious disease in children. Pediatr Infect Dis J. 2008;27:618–22. 10.1097/INF.0b013e31816b1947 18520970

[11] Jelinek T, Loscher T. Clinical features and epidemiology of tick typhus in travelers. J Travel Med. 2001;8:57–9. 11285163 10.2310/7060.2001.24485

[12] Cazorla C, Socolovschi C, Jensenius M, Parola P. Tick-borne diseases: spotted fever group rickettsioses in Africa. Infect Dis Clin North Am. 2008;22:531–44. 10.1016/j.idc.2008.03.009 18755389

[13] Raoult D, Fournier PE, Fenollar F, Jensenius M, Prioe T, de Pina JJ, *Rickettsia africae*, a tick-borne pathogen in travelers to sub-Saharan Africa. N Engl J Med. 2001;344:1504–10. 10.1056/NEJM200105173442003 11357153

[14] Jensenius M, Fournier PE, Vene S, Hoel T, Hasle G, Henriksen AZ, African tick bite fever in travelers to rural sub-Equatorial Africa. Clin Infect Dis. 2003;36:1411–7. 10.1086/375083 12766836

[15] Ding T, Lloyd G, Tolley H, Bradlow A. Tick bite fever and arthritis associated with travel to Africa. Ann Rheum Dis. 2004;63:1703–4. 10.1136/ard.2003.019752 15547103 PMC1754867

[16] Jensenius M, Fournier PE, Fladby T, Hellum KB, Hagen T, Priø T, Sub-acute neuropathy in patients with African tick bite fever. Scand J Infect Dis. 2006;38:114–8. 10.1080/00365540500321579 16449002

[17] Roch N, Epaulard O, Pelloux I, Pavese P, Brion JP, Raoult D, African tick bite fever in elderly patients: 8 cases in French tourists returning from South Africa. Clin Infect Dis. 2008;47:e28–35. 10.1086/589868 18558881

[18] Azuma M, Nishioka Y, Ogawa M, Takasaki T, Sone S, Uchiyama T. Murine typhus from Vietnam, imported into Japan. Emerg Infect Dis. 2006;12:1466–8. 17073110 10.3201/eid1209.060071 PMC3294741

[19] Parola P, Vogelaers D, Roure C, Janbon F, Raoult D. Murine typhus in travelers returning from Indonesia. Emerg Infect Dis. 1998;4:677–80. 10.3201/eid0404.980423 9866749 PMC2640266

[20] Pether JV, Jones W, Lloyd G, Rutter DA, Barry M. Fatal murine typhus from Spain. Lancet. 1994;344:897–8. 10.1016/S0140-6736(94)92875-4 7916435

[21] Jensenius M, Montelius R, Berild D, Vene S. Scrub typhus imported to Scandinavia. Scand J Infect Dis. 2006;38:200–2. 10.1080/00365540500277342 16500780

[22] Watt G, Strickman D. Life-threatening scrub typhus in a traveler returning from Thailand. Clin Infect Dis. 1994;18:624–6. 8038320 10.1093/clinids/18.4.624

[23] Bakken JS, Dumler S. Human granulocytic anaplasmosis. [viii. ]. Infect Dis Clin North Am. 2008;22:433–48. 10.1016/j.idc.2008.03.011 18755383

[24] Laferl H, Hogrefe W, Kock T, Pichler H. A further case of acute human granulocytic ehrlichiosis in Slovenia. Eur J Clin Microbiol Infect Dis. 1999;18:385–6. 10.1007/PL00015026 10421051

[25] van Dobbenburgh A, van Dam AP, Fikrig E. Human granulocytic ehrlichiosis in western Europe. N Engl J Med. 1999;340:1214–6. 10.1056/NEJM199904153401517 10206853

[26] Ta TH, Jiménez B, Navarro M, Meije Y, González FJ, Lopéz-Veléz R. Q fever in returned febrile travelers. J Travel Med. 2008;15:126–9. 10.1111/j.1708-8305.2008.00191.x 18346248

[27] Potasman I, Rzotkiewicz S, Pick N, Keysary A. Outbreak of Q fever following a safari trip. Clin Infect Dis. 2000;30:214–5. 10.1086/313613 10619763

[28] Isaksson HJ, Hrafnkelsson J, Hilmarsdóttir I. Acute Q fever: a cause of fatal hepatitis in an Icelandic traveler. Scand J Infect Dis. 2001;33:314–5. 10.1080/003655401300077441 11345226

[29] Matteelli A, Castelli F, Spinetti A, Bonetti F, Graifenberghi S, Carosi G. Short report: verruga peruana in an Italian traveler from Peru. Am J Trop Med Hyg. 1994;50:143–4. 8116804 10.4269/ajtmh.1994.50.143

[30] Lydy SL, Eremeeva ME, Asnis D, Paddock CD, Nicholson WL, Silverman DJ, Isolation and characterization of *Bartonella bacilliformis* from an expatriate Ecuadorian. J Clin Microbiol. 2008;46:627–37. 10.1128/JCM.01207-07 18094131 PMC2238110

[31] Florin TA, Zaoutis TE, Zaoutis LB. Beyond cat scratch disease: widening spectrum of *Bartonella henselae* infection. Pediatrics. 2008;121:e1413–25. 10.1542/peds.2007-1897 18443019

[32] Jensenius M, Fournier PE, Hellum KB, Wesslén L, Caruso G, Priø T, Sequential changes of hematological and biochemical parameters in African tick bite fever. Clin Microbiol Infect. 2003;9:678–83. 10.1046/j.1469-0691.2003.00713.x 12925109

[33] Fournier PE, Jensenius M, Laferl H, Vene S, Raoult D. Kinetics of antibody responses in *Rickettsia africae* and *Rickettsia conorii* infections. Clin Diagn Lab Immunol. 2002;9:324–8. 11874871 10.1128/CDLI.9.2.324-328.2002 PMC119950

[34] Jensenius M, Fournier PE, Vene S, Ringertz SH, Myrvang B, Raoult D. Comparison of immunofluorescence assay, Western blotting and cross-adsorption for the diagnosis of African tick bite fever. Clin Diagn Lab Immunol. 2004;11:768–83.Medline10.1128/CDLI.11.4.786-788.2004 PMC440600 15242958

[35] Rolain JM, Brouqui P, Koehler JE, Maguina C, Dolan MJ, Raoult D. Recommendations for treatment of human infections caused by *Bartonella* species. Antimicrob Agents Chemother. 2004;48:1921–33. 10.1128/AAC.48.6.1921-1933.2004 15155180 PMC415619

